# Improvement of Asia-Pacific colorectal screening score and evaluation of its use combined with fecal immunochemical test

**DOI:** 10.1186/s12876-019-1146-2

**Published:** 2019-12-27

**Authors:** Xu-xia He, Si-yi Yuan, Wen-bin Li, Hong Yang, Wen Ji, Zhi-qiang Wang, Jian-yu Hao, Chuan Chen, Wei-qing Chen, Ying-xin Gao, Ling-bo Li, Kai-liang Cheng, Jia-ming Qian, Li Wang, Jing-nan Li

**Affiliations:** 1 Department of Gastroenterology, Key Laboratory of Gut Microbiota Translational Medicine Research, Chinese Academy of Medical Science, Peking Union Medical College Hospital, No.1, Shuaifuyuan, Dongcheng District, Beijing, 100730 China; 2 Department of Gastroenterology, Chongqing Cancer Hospital, Chongqing, 400030 China; 3 Second Medical Center, Chinese PLA General Hospital, Beijing, 100853 China; 4 Department of Gastroenterology, Beijing Chao-Yang Hospital, Beijing, 100020 China; 5 Department of Gastroenterology, University of Hong Kong-Shenzhen Hospital, Guangdong Province, Shenzhen, 518053 China; 6 Medical School of Nantong University, Nantong, Jiangsu Province, 226001 China; 7 Department of Epidemiology and Biostatistics, Institute of Basic Medical Sciences Chinese Academy of Medical Sciences, School of Basic Medicine Peking Union Medical College, Beijing, 100005 China

**Keywords:** Colorectal cancer screening, Advanced colorectal neoplasia, Predictive model, Fecal immunochemical test, High risk population

## Abstract

**Background:**

The Asia-Pacific Colorectal Screening (APCS) score is effective to screen high-risk groups of advanced colorectal neoplasia (ACN) patients but needs revising and can be combined with the fecal immunochemical test (FIT). This paper aimed to improve the APCS score and evaluate its use with the FIT in stratifying the risk of ACN.

**Methods:**

This prospective and multicenter study enrolled 955 and 1201 asymptomatic Chinese participants to form the derivation and validation set, respectively. Participants received the risk factor questionnaire, colonoscopy and FIT. Multiple logistic regression was applied, and C-statistic, sensitivity and negative predictive values (NPVs) were used to compare the screening efficiency.

**Results:**

A modified model was developed incorporating age, body mass index (BMI), family history, diabetes, smoking and drinking as risk factors, stratifying subjects into average risk (AR) or high risk (HR). In the validation set, the HR tier group had a 3.4-fold (95% CI 1.8–6.4) increased risk for ACN. The C-statistic for the modified score was 0.69 ± 0.04, and 0.67 ± 0.04 for the original score. The sensitivity of the modified APCS score combined with FIT for screening ACN high-risk cohorts was 76.7% compared with 36.7% of FIT alone and 70.0% of the modified APCS score alone. The NPVs of the modified score combined with FIT for ACN were 98.0% compared with 97.0% of FIT alone and 97.9% of the modified APCS score alone.

**Conclusions:**

The modified score and its use with the FIT are efficient in selecting the HR group from a Chinese asymptomatic population.

## Background

Colorectal cancer (CRC) is the fourth leading malignant tumor for incidence and the second most common for mortality in both sexes across the world based on the 2018 GLOBOCAN estimates [1], and the morbidity of CRC in the Chinese population has continued to rise in recent years [2]. CRC screening, including the fecal occult blood test (FOBT) and colonoscopy, has been shown to effectively reduce the prevalence of colorectal neoplasia and increase survival [3–5]. Guidelines generally recommend that CRC screening should be carried out in people aged over 50 years via FOBT, including guaiac­based (gFIT) and immunochemical (FIT), or endoscopy, including colonoscopy and sigmoidoscopy [6], which is hindered to some extent in China due to the large population and limited medical resources. It is reported that the FIT is relatively cost-effective and annual FIT can reduce CRC mortality by approximately 30% [7, 8], but the effect is achieved in prerequisite that positive FIT be followed by more expensive tests, including colonoscopy. Therefore, it is important to develop a simple predictive model that can be used to screen high-risk (HR) groups of CRCs to make subsequent screening methods, mainly colonoscopy, more effective and targeted. Moreover, due to the cost-effectiveness of FIT, the risk score can be combined with FIT to achieve a higher level of screening efficiency.

The Asia-Pacific Colorectal Screening (APCS) score was established via the study of a derivation set of 860 subjects and a validation set of 1892 subjects [9]. Nevertheless, the original APCS model is relatively simple and is based merely on elementary clinical data, including sex, age, family history and smoking status. Other risk factors, such as diabetes mellitus [10], alcohol consumption [11] and obesity [12], were overlooked, although they have been reported to be closely related to CRC in previous studies. In addition, the original APCS score overlooked the significance of FIT in current CRC screening and failed to test the combination of the score with FIT. Therefore, our team hoped to further improve the original APCS score model by adding other risk factors to make it more comprehensive and effective and investigate the validity of the combined use of the modified score and FIT.

Based on a list of CRC-related risk factors, including sex, age, smoking status, alcohol consumption, diabetes, family history, obesity, diet and exercise, this study aimed to improve the existing APCS score and validate the modified model to confirm its effectiveness in screening high-risk groups of CRC among a Chinese asymptomatic population and further test the validity of the score combined with FIT.

## Methods

### Study aims and funding

The purpose of this study was three-fold. First, it aimed to improve the original APCS score as a risk-prediction model for ACN screening in an asymptomatic Chinese population. Second, it was intended to validate the effectiveness of the modified score for clinical use in a larger population and third, to compare its efficiency with FIT for screening ACN high-risk cohorts. This study was supported by the National Natural Science Foundation of China (Grant No. 81770559, 81,370,500) and Chinese Academy of Medical Sciences Innovation Fund for Medical Sciences (2016-12 M-3-001).

### Study participants

We prospectively enrolled 955 participants to form the derivation set in 3 hospitals (Peking Union Medical College Hospital, Beijing Chaoyang Hospital and Beijing Friendship Hospital) from September 2016 to December 2017. Then, a modified screening score can be conducted based on the analysis of data from the derivation set. Afterwards, 1201 participants were enrolled in the same way into the validation set in 5 hospitals (Peking Union Medical College Hospital, Chongqing Cancer Hospital, Chinese PLA General Hospital, Beijing Chaoyang Hospital and The University of Hong Kong-Shenzhen Hospital) from January 2018 to December 2018. All participants were outpatients over 40 years old who were asymptomatic and agreed to join the study. Those with a medical history of colorectal cancer, colorectal polyps or inflammatory bowel disease, or with colonoscopy contraindications were excluded.

### Study design, ethical approval and consent to participate

This prospective, multicenter, large-scale study was conducted in asymptomatic Chinese subjects based on questionnaires, FIT, colonoscopy findings and statistical analysis. This study was approved by the Ethical Committee of Peking Union Medical College Hospital, and the registration number on “Chinese Clinical Trial Registry” was ChiCTR-SOD-16008774. All participants signed informed consent before enrollment in this study.

### Questionnaire and colonoscopy

All participants in the derivation set and the validation set filled out the risk factor questionnaire and underwent colonoscopy. Considering the possible risk factors of ACN, we designed the questionnaire to collect basic demographic variables and certain personal information incorporating age, sex, height, weight, family history of CRC in a first-degree relative, smoking status, alcohol consumption, diet preference, exercise habits, and diabetes mellitus. Questionnaires were made simple to boost patient cooperation as much as possible. Alcohol consumption was defined as an intake of 150 g ethanol or more per week. Regular exercise required working out at least 3 times per week and 30 min every time. Height, weight and diabetes mellitus were self-reported. Diet preference was defined as vegetable-based, meat-based, or mixed food. Smoking was defined as an active smoking history of more than 100 cigarettes and a smoking history in the past 1 year. All questionnaires were completed by eligible subjects with the assistance of medical staff and trained volunteers.

Standardized colonoscopy was conducted by experienced endoscopists in a double-blinded way at all study sites. The withdrawal observation time was ≥6 min to minimize the possibility of false negative results under the requirements of the international standard of quality assurance for colonoscopy procedures. Colorectal neoplasia includes non-advanced adenoma and advanced colorectal neoplasia (ACN). ACN was defined as CRC or advanced adenoma. Advanced adenoma was defined as adenomas ≥10 mm in diameter, villous histological features (at least 25% villous), high-grade dysplasia, or any combination thereof.

Additionally, FIT (WHPM Bioresearch and Technology Co., Ltd) was conducted in part of the validation cohort for better evaluating the screening efficiency for ACN in combination with the modified risk score. The cutoff value of 0.2 μg Hb/mL feces was defined for FIT positivity in our study. Of note, only part of the validation set received FIT because not all hospitals adopted FIT and part of the outpatient participants failed to offer stool samples.

### Sample size estimation

In our study, the prevalence of ACN in the derivation set was 4.1%. The reported prevalence of ACN in Asia was between 3 and 12% [13–15]. According to these data, at least 1200 asymptomatic subjects were needed in the validation set to achieve the power of 80% at *p* < 0.05.

### Statistical analysis

All statistical analyses were conducted with SPSS software (version 25.0). Percentages were reported as proportions and 95% confidence intervals (CIs). Continuous variables were expressed as the mean ± standard deviation (SD). The statistical review of the study was performed by a biomedical statistician.

Risk factors were analyzed by the Pearson chi-square test. The risk factors with a *P* value of ≤0.15 were selected for multivariate logistic regression analysis. The risk score was developed through the analysis of odds ratio (OR), the Hosmer-Lemeshow goodness-of-fit statistic, the C-statistic and area under receiver operating characteristic (ROC). The sensitivity and negative predictive values (NPVs) of the modified APCS score combined with FIT for screening high-risk cohorts for colonoscopy were thereafter compared. The number needed to screen (NNS) for detecting one advanced neoplasia was calculated.

## Results

### Characteristics of the participants

**Fig. 1:**
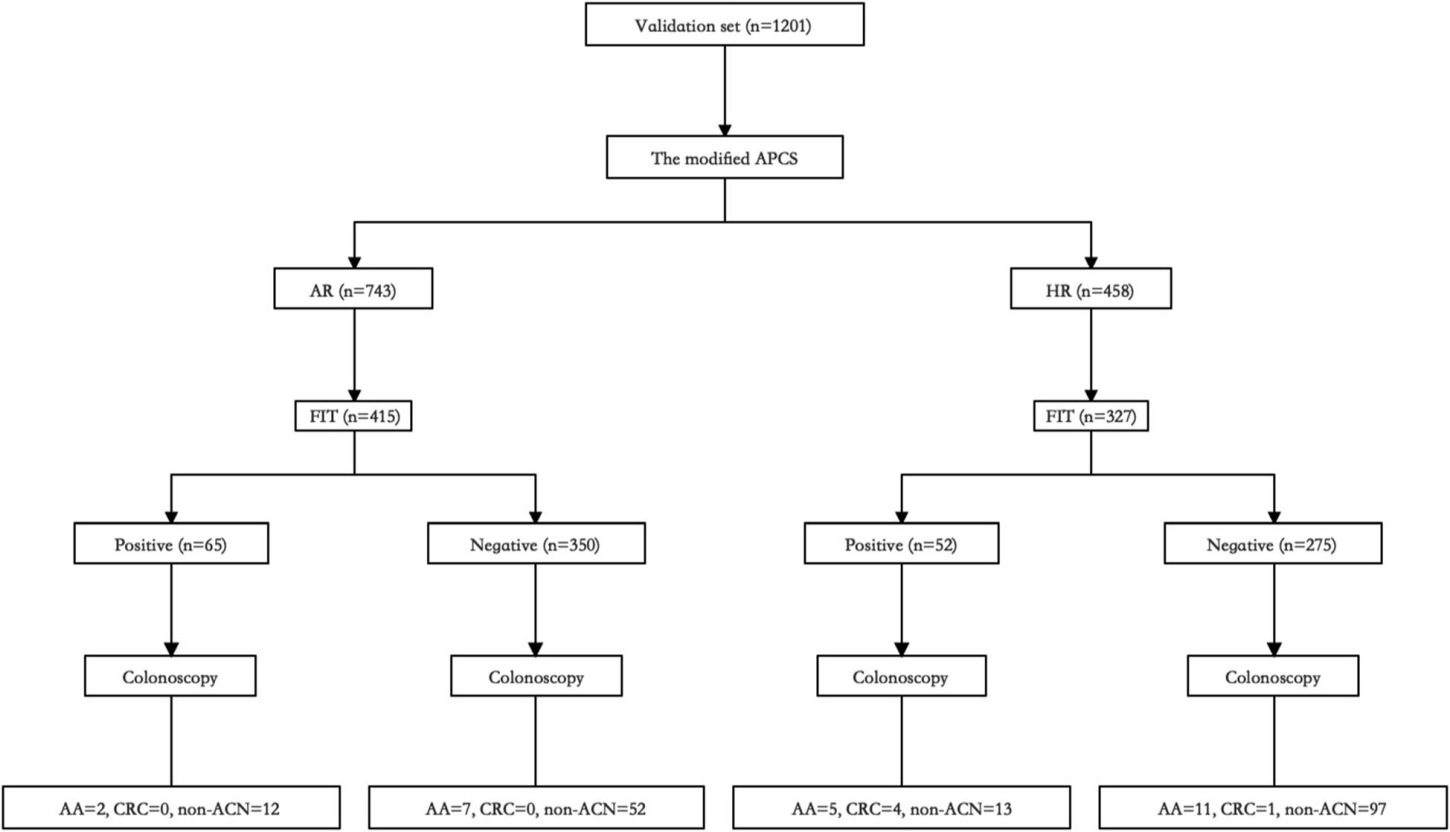
Flowchart demonstrating the process for the validation set. APCS: the Asia-Pacific Colorectal Screening score. AR: average risk. HR: high risk. FIT: fecal immunochemical test. AA: advanced adenoma. ACN: advanced colorectal neoplasia. Non-ACN: non- advanced colorectal neoplasia. CRC: colorectal cancer

The derivation set and the validation set comprised 955 and 1201 participants, respectively (Table 1). A total of 197 (20.6%) subjects in the derivation set were diagnosed with colorectal neoplasia by colonoscopy, including 34 (3.6%) cases of advanced adenoma and 5 (0.5%) cases of colorectal cancers. Moreover, the validation set was composed of 383 (31.9%) cases of colorectal neoplasia, including 35 (2.9%) cases of advanced adenoma and 10 (0.8%) cases of cancers. The baseline prevalence of ACN in the two sets was 4.1 and 3.7%, respectively. In terms of FIT, there were a total of 742 subjects that received the test, including 117 (15.8%) positive cases. The distribution of all these factors in the two cohorts is shown in Table 1, and Fig. 1 is a flowchart demonstrating the process of the validation set.

**Table 1:** Characteristics of the derivation and validation cohorts

	Derivation cohort *n* = 955	Validation cohort *n* = 1201
Age, y, median ± SD	53.5 ± 8.3	50.7 ± 11.4
Sex, male, n (%)	518 (54.2)	743 (61.9)
BMI, kg/m^2^, median ± SD	24.3 ± 3.6	24.6 ± 4.3
Current or ex-smoking, n (%)	303 (31.7)	370 (30.8)
Alcohol consumption, n (%)	452 (47.3)	391 (32.6)
Diabetes, n (%)	38 (4.0)	128 (10.7)
Family history, n (%)	87 (9.1)	71 (5.9)
Diet, n (%)		NA
Vegetable	333 (34.9)	
Meat	187 (19.6)	
Unspecific	435 (45.5)	
Exercise, little, n (%)	833 (87.2)	NA
Fecal immunochemical test, n (%)	NA	742^a^
Positive		117 (15.8)
Negative		625 (84.2)
Colorectal neoplasia, n (%)	197 (20.6)	383 (31.9)
Colorectal cancer, n (%)	5 (0.5)	10 (0.8)
Advanced neoplasia, n (%)	39 (4.1)	45 (3.7)

*NA* Not applicable, *SD* Standard deviation, *BMI* Body mass index

^a^Only 742 subjects in the validation set received the fecal immunochemical test due to practical factors

### Univariate and multivariate predictors of colorectal neoplasia and advanced neoplasia in the derivation cohort

We performed univariate and multivariate analyses for each risk factor in the derivation set (Table 2). For ACN, age (59–69 or ≥ 70 years), smoking, alcohol consumption, diabetes, BMI (≥23 [16]) and family history of CRC in a first-degree relative were significant factors, with ORs (95% CI) of 1.4 (0.7–3.0) or 5.2 (1.8–15.1), 2.4 (1.2–4.5), 2.0 (1.0–4.0), 3.9 (1.5–10.7), 2.2 (1.0–4.8), and 2.3 (1.0–5.4), respectively. Further, in multivariate analyses, these factors exhibited ORs (95% CI) of 2.9 (1.4–6.1) or 9.8 (3.4–28.2), 1.8 (0.8–3.8), 2.1 (0.9–4.9), 3.5 (1.2–9.9), 2.1 (0.9–4.9), and 2.5 (1.0–6.1), respectively (Table 2). The Hosmer-Lemeshow goodness-of-fit statistic was *p* = 0.93 for the derivation cohort.

**Table 2:** The relationship between various risk factors and advanced colorectal neoplasia

Risk factors	Unadjusted		Adjusted			
	OR (95% CI)	*P* value	β coefficient	SE	OR (95% CI)	*P* value
Sex	1.5 (0.8–3.0)	0.207	–	–	–	–
Age						
40–58	Reference		–	–	Reference	
59–69	1.4 (0.7–3.0)	0.081	1.062	0.384	2.9 (1.4–6.1)	0.006
≥ 70	5.2 (1.8–15.1)	0.001	2.278	0.542	9.8 (3.4–28.2)	< 0.001
Family History	2.3 (1.0–5.4)	0.050	0.921	0.454	2.5 (1.0–6.1)	0.043
Smoking	2.4 (1.2–4.5)	0.007	0.572	0.395	1.8 (0.8–3.8)	0.148
Alcohol consumption	2.0 (1.0–4.0)	0.032	0.744	0.429	2.1 (0.9–4.9)	0.083
Diabetes	3.9 (1.5–10.7)	0.004	1.242	0.536	3.5 (1.2–9.9)	0.020
BMI ≥ 23	2.2 (1.0–4.8)	0.046	0.759	0.422	2.1 (0.9–4.9)	0.072
BMI ≥ 25	1.4 (0.8–2.7)	0.262	–	–	–	–
BMI ≥ 28	0.8 (0.3–2.4)	0.756	–	–	–	–
Diet			–	–	–	–
Vegetable	1.2 (0.6–2.3)	0.631	–	–	–	–
Meat	1.1 (0.5–2.4)	0.881	–	–	–	–
Exercise	0.9 (0.4–2.4)	0.868	–	–	–	–

*OR* Odds ratio, *SE* Standard error, *CI* Confidence interval, *BMI* Body mass index

### Development of the risk score

We incorporated age, family history of first-degree relatives, smoking, alcohol consumption, diabetes and BMI into the risk score model for ACN with points weighted based on adjusted ORs from the multiple logistic regression. Each adjusted OR was rounded to appropriate integers to simplify the score as much as possible and increase the feasibility.

The final score model is shown in Table 3. The total score of the risk model is 12, with points assigned as follows: age < 59 (0), 59–69 (2), or ≥ 70 (5); family history absent (0) or present (2); never smoked (0) or current or past smoker (1); never drank (0) or current or past drinker (1); diabetes absent (0) or present (2); BMI < 23 (0) or ≥ 23 (1). Through a comprehensive analysis of the ROC curve, Youden’s index and Euclidean’s index, we artificially defined two risk tiers: 0–2 as average risk (AR), and ≥ 3 as high-risk (HR). Among the derivation cohort, 541 (56.6%) and 414 (43.4%) were in the AR and HR tiers, respectively (Table 4). The C-statistic in the derivation set was 0.74 ± 0.04.

**Table 3:** The final risk scores for screening of advanced colorectal neoplasia in Chinese population

Risk Factors	Criteria	Points
Age	<59	0
	59–69	2
	≥70	5
Family history	Absent	0
	Present	2
Smoking	Never	0
	Current or past	1
Drinking	Never	0
	Current or past	1
Diabetes	Absent	0
	Present	2
BMI	< 23	0
	≥ 23	1

*BMI* Body mass index

**Table 4:** Stratification of the prevalence of advanced colorectal neoplasia by risk tier

Risk tier	Derivation set		Validation set						
	Number (%)	ACN (%) (95% CI)	Number (95% CI)	ACN (%) (95% CI)	OR (95% CI)	Neoplasia (%) (95% CI)	OR (95% CI)	CRC (%) (95% CI)	OR (95% CI)
AR (0–2)	541 (56.6)	10 (1.8) (0.7–3.0)	743 (61.9)	15 (2.0) (1.0–3.0)	–	167 (22.5) (19.5–25.5)	–	3 (0.4) (0–0.9)	–
HR (≥3)	414 (43.4)	29 (7.0) (4.5–9.5)	458 (38.1)	30 (6.6) (4.3–8.8)	3.4 (1.8–6.4)	216 (47.2) (42.6–51.8)	3.1 (2.4–4.0)	7 (1.5) (0.4–2.7)	3.8 (1.0–14.9)
Total	955 (100)	39 (4.1) (2.8–5.1)	1201 (100)	45 (3.7) (2.7–4.8)	–	383 (31.9) (29.3–34.5)	–	10 (0.8) (0.3–1.3)	–

*ACN* Advanced colorectal neoplasia, *OR* Odds ratio, *CI* Confidence interval. *AR* Average risk, *HR* High risk

### Validation of the risk score model

Based on the adjusted risk score, 743 (61.9%) subjects in the validation set were classified as the AR tier (score 0–2), while 458 (38.1%) were classified as the HR tier (score ≥ 3). The prevalence of ACN in the AR and HR categories were 2.0% (95% CI 1.0–3.0%) and 6.6% (95% CI 4.3–8.8%), respectively (*P* < 0.001). The HR cohort showed a 3.4-fold (95% CI 1.8–6.4) increased risk compared to the AR cohort for ACN, and similarly, 3.1 (95% CI 2.4–4.0) for colorectal neoplasia and 3.8 (95% CI 1.0–14.9) for CRC (Table 4). The C-statistic for the modified score predicting ACN was 0.69 ± 0.04, compared with 0.67 ± 0.04 for the original APCS score. The Hosmer-Lemeshow goodness-of-fit statistic was 0.87 in the validation cohort. Furthermore, NNS for detecting one advanced neoplasia was 23.

### Comparison with FIT

In the validation set, a total of 742 subjects received FIT, and the distribution of FIT results combined with the above risk score tiers are listed in Table 5. The combined risk score and FIT positive screening were defined as FIT positive or classified into HR score tier; the combined screening negative was defined as FIT negative in AR group. Thus, the sensitivity of the modified APCS score combined with FIT for screening ACN high-risk cohorts for colonoscopy was 76.7% compared with 36.7% of FIT alone and 70.0% of the modified score alone, indicating that the screening efficiency of the combined method was better than that of the score or FIT alone. The tendency was similar for advanced adenoma, CRC, and non-advanced neoplasia screening. Moreover, NPVs of the modified score combined with FIT were 98.0% compared with 97.0% of FIT alone and 97.9% of the modified APCS score alone, with the slight difference similar for other categories of colorectal diseases (Table 5).

**Table 5:** Fecal immunochemical test distribution in the validation set

	AR (0–2)			HR (≥3)			Se-1^a^ (%)	Se-2^a^ (%)	Se-3^a^ (%)	NPV −1^b^ (%)	NPV −2^b^ (%)	NPV −3^b^ (%)
	n (%)	FIT-	FIT+	n (%)	FIT-	FIT+						
ACN	9 (2.2)	7	2	21 (6.4)	12	9	76.7%	36.7%	70.0%	98.0%	97.0%	97.9%
Advanced adenoma	9 (2.2)	7	2	16 (4.9)	11	5	72.0%	28.0%	64.0%	98.0%	97.2%	97.9%
CRC	0 (0)	0	0	5 (1.5)	1	4	100.0%	80.0%	100.0%	100.0%	99.8%	100.0%
Nonadvanced neoplasia	64 (15.4)	52	12	110 (33.6)	97	13	70.1%	14.4%	63.2%	85.4%	76.9%	84.9%
Negative	342 (82.4)	291	51	196 (59.9)	166	30						
Total	415 (100)	350	65	327 (100)	275	52						

*ACN* Advanced colorectal neoplasia, *CRC* Colorectal cancer, *AR* Average risk, *HR* High risk, *FIT* Fecal immunochemical test, *NPV* Negative predictive value

^a^Se-1~3: sensitivity of the modified APCS score combined with fecal immunochemical test (Se-1), of fecal immunochemical test alone (Se-2), and of the modified APCS score alone (Se-3) for screening high risk cohorts for colonoscopy

^b^NPV-1~3: NPV of the modified APCS score combined with fecal immunochemical test (NPV-1), of fecal immunochemical test alone (NPV-2), and of the modified APCS score alone (NPV-3) for screening high risk cohorts for colonoscopy

## Discussion

Effective screening methods, principally referring to FIT and colonoscopy, can reduce the prevalence and mortality of CRCs. However, standardized and effective CRC screening strategies cannot be carried out across China, mainly due to resource limitations and lack of health awareness, leading to the urgency for more efficient and targeted primary screening schedules. Under such conditions, the economical and simple risk score system or its combined use with FIT are attracting an increasing level of interest in the world. Some studies [17–19] have attempted to establish a screening questionnaire model for a high-risk group of CRCs that is generally not comprehensive and short of feasibility. The APCS system established by scholars from the Chinese University of Hong Kong [9] considered risk factors including sex, age, family history and smoking status and has been validated by different studies in various parts of Asia-Pacific area and even in Western countries [20–28]. However, the APCS score failed to collect information on the height and weight of participants, so it was impossible to judge the role of BMI in the prediction system. In addition, the original score failed to take FIT, which is also cost-effective and validated in current CRC screening guidelines, into account.

In our study, risk factors related to colorectal neoplasia were extensively reviewed and evaluated to establish a modified screening model for CRC. The prevalence of ACN in the derivation set and the validation set were 4.1 and 3.7%, respectively, which were generally consistent with previous findings [9, 13–15, 28]. In previous research, the contributions of age, gender, smoking, drinking, family history, diabetes, diet and exercise [10, 17–19, 29–32] have been recognized as major risk factors for CRC. Based on data analysis in this study, the final risk score incorporated age, family history, smoking, alcohol consumption, diabetes and BMI as factors (Table 3). According to this modified score, the HR cohort in the validation set exhibited a 3.4-fold (95% CI 1.8–6.4) increased risk than the AR cohort for ACN. The C-statistic for the modified risk score was 0.69 ± 0.04, slightly higher than that for the original APCS system. Overall, the modified APCS score showed good discrimination and screening effect through data validation. Notably, our modified score assigned a factor age ≥ 70 with five points, which was higher than the original APCS score, which assigned an age of 50–69 with two points and an age of ≥70 with three points, and our study defined two tiers of risk instead of the three in the original score. The weight of factors came from the univariate and multivariate data analysis of our study, and the cut-off value of the two risk tiers was defined via the analysis of the ROC curve in convenience of developing a practical CRC screening strategy in China. Our modified score recommends that an asymptomatic subject is categorized as a high-risk tier for CRC if he is ≥70 years old or has one of these factors, including 59–69 years old, a CRC family history and a past history of diabetes mellitus with any other risk factors, or is overweight with a habit of smoking and drinking. Overall, the modified score is more efficient and simpler than the original score. For example, based on the original score, a patient ≥70 years of age is assigned 3 points and defined as moderate risk (2–3 points), but it is actually difficult to decide for this individual whether to undergo further screening. In contrast, the two tiers of risk avoid such embarrassment in regard to practical implementation.

Compared to the original score, we introduced diabetes as a key risk factor with 2 points, alcohol consumption with 1 point and BMI with 1 point. In addition, sex and other targeted risk factors were left out of our modified score because sex showed significance only for colorectal neoplasia but not for ACN, and other factors remained nonsignificant for both. The incorporation of diabetes and alcohol consumption into the score system was in accordance with previous study results [10, 11]. BMI has long been recognized as a risk factor for CRC [12], and current studies suggest that there is a moderate association between general obesity (as determined by BMI) and the incidence and mortality of CRC and colorectal adenoma [33]. Similarly, several studies have recently worked on modifying the APCS score by adding BMI for the prediction of advanced neoplasia and reported satisfying discrimination efficiency outcomes [23, 34, 35]. Therefore, it is reasonable to incorporate BMI into the CRC prediction and screening questionnaire. Of note, a BMI cutoff of≥23 kg/m^2^ was adopted in this study as a risk factor by logistic regression analysis. We suggest this is reasonable because generally Asian population tend to be thinner and current guidelines in China accepted BMI 24.0–27.9 kg/m^2^ as overweight, and some studies even adopted BMI 23.0–27.9 kg/m^2^ as the definition of overweight [36]. Besides, it is reported that for BMI over 25.0 kg/m^2^, mortality increased approximately log-linearly with BMI, and the hazard ratio per 5 kg/m^2^ units higher BMI was 1.39 (1.34–1.44) in east Asia [37].

Sex is another definite risk factor for CRC, but it failed to show a significant difference in our modeling process. It has been reported that men have an increased risk for advanced neoplasia compared with women, and this positive association was significant across all age groups from age 40 to older than 70 years [38], but it was affected by cancer site, diet, and menopausal status [39]. The confounding influence may account for the failure of adding sex into our new score model. Similar situations exist with the association of diet or exercise with ACN. Therefore, it is advisable to detail the questionnaire items and unify the evaluation criteria to further test the relationship of sex, diet, and exercise with the occurrence of ACN.

**Fig. 2:**
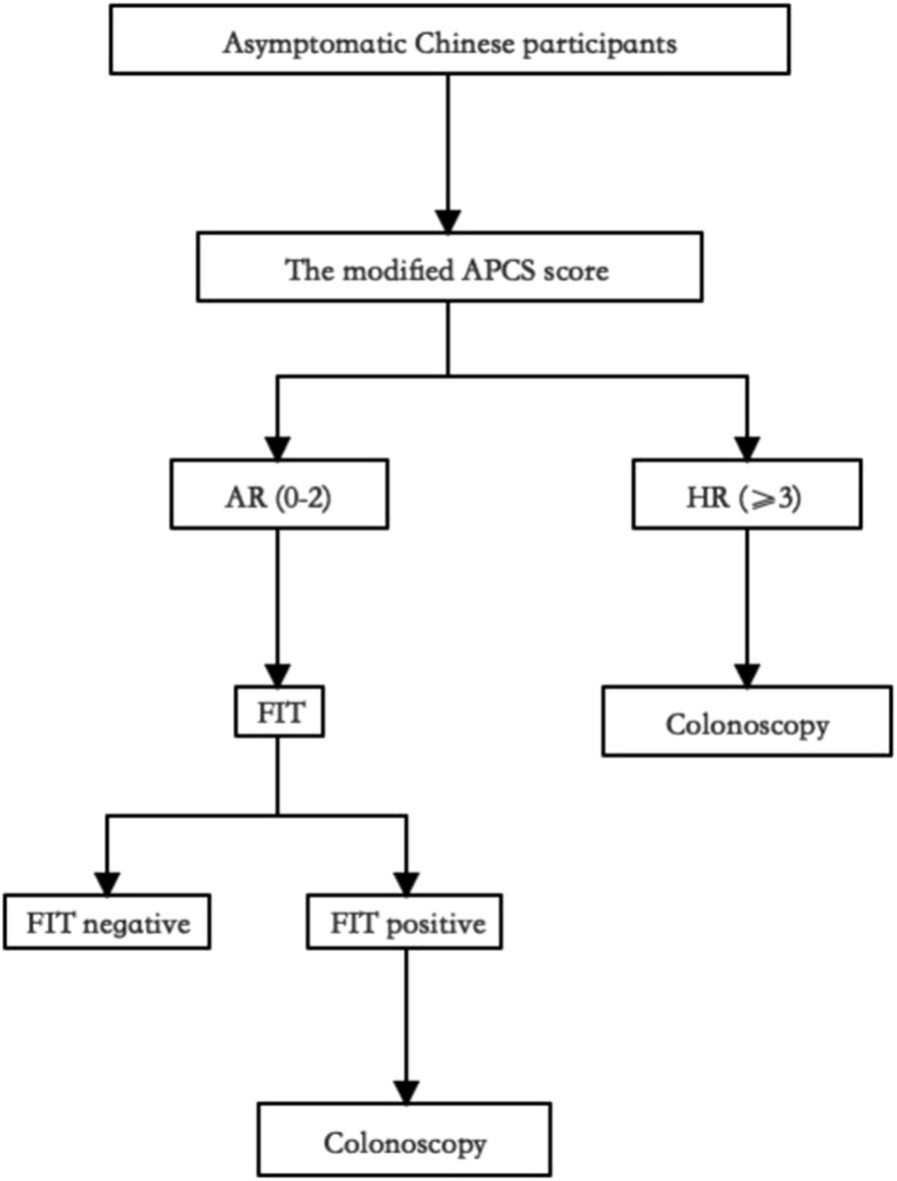
Conceptual colorectal cancer screening algorithm of APCS score combined with FIT. APCS: the Asia-Pacific Colorectal Screening score. AR: average risk. HR: high risk. FIT: fecal immunochemical test

In recent years, studies have been investigating the combined use of the prediction score with FIT for raising the screening efficiency of the high-risk cohort for ACN. As reported, the combined use of the APCS score and FIT could correctly instruct 70.6% subjects with ACN and 95.1% subjects with CRC for early colonoscopy examination, thus substantially optimizing medical resources [40]. In our study, the sensitivity and NPVs of combining the modified score with FIT for predicting ACN were higher than those of using either alone, and the combination of these two economical and simple tests can detect the majority of ACN cases as well as CRC. Thus, in our conceptual CRC screening algorithm (Fig. 2), the asymptomatic Chinese subjects should first fill out the risk score questionnaire, and those categorized as AR tiers are then referred for FIT. Finally, colonoscopy is highly recommended for those in the HR tier or in the AR tier but with positive FIT. The entire screening path can triage people for colonoscopy in a more effective way than the traditional screening strategy, which reduces the workload and optimizes the medical resources of ACN screening for clinical practitioners and government policymakers, especially in vast developing countries such as China.

Some limitations exist in this study. First, most of the data were collected through questionnaire by means of self-reporting, so it is possible to misestimate some risk factors. For example, for the risk factor for diabetes, patients with self-knowledge of diabetes may be lower than the actual prevalence of diabetes, and the estimate of diet may be quite subjective, resulting in bias. Second, though the whole screening path combing FIT and the screening score showed good discrimination in triaging HR population for ACN, the C-statistic for the modified risk score was only slightly higher than that for the original APCS system, which may be attributed to confounding factors that lowered the efficacy. Except for the targeted risk factors of CRC, we basically did not compare more detailed information, such as cancer site, pathological types, or quantity of alcohol intake or collected other population characteristics such as menopause status in women and cholecystectomy history. Third, the participants in the derivation and validation cohorts mainly came from hospitals in Beijing and Chongqing without covering other parts of China and even other countries in the Asia-Pacific area. Fourth, due to practical influence, only part of the validation cohort (742 out of 1201 participants) received FIT, partially causing selection bias in evaluating the efficiency of the combined use of the modified score and FIT. Therefore, further investigation is needed to make the primary screening of ACN in China more precise and practical.

## Conclusions

In summary, our study established a modified APCS score based on age, BMI, family history, diabetes, smoking and drinking and indicated that its use combined with FIT is efficient in selecting a high-risk group for CRC from a Chinese asymptomatic population to decrease the workload and optimize the resources of CRC screening for clinical practitioners and government policymakers.

## Data Availability

The datasets generated and analyzed during the current study are available from the corresponding author on reasonable request.
